# Editorial: Gluten: yes, no, maybe

**DOI:** 10.3389/fmed.2023.1225139

**Published:** 2023-06-09

**Authors:** Stefano Guandalini

**Affiliations:** Department of Pediatrics, University of Chicago Medicine, Chicago, IL, United States

**Keywords:** gluten yes, no, maybe, gluten, celiac disease, non-celiac wheat sensitivity (NCWS), NRCD

The role of gluten in human disorders has become one of the hottest topics, not only within the scientific community, where it often generates heated, opinions-loaded, debates but also in the general public, with a myriad of self-proclaimed experts voicing their points of view.

To tackle some aspects of this immense and ever-expanding world, we opted for inviting a few but extremely well-qualified investigators in this field to offer us not a comprehensive review of such a wide topic but rather advanced data ranging from sophisticated *in vitro* research on the effects of a post-biotic (post-biotics being the new child in town in the world of probiotics) on the inflammation caused by gliadin peptides, all the way to new prospects of treatment such as evaluating the potential use of ancient grains in the diet of individuals presenting the so-called “non-celiac wheat sensitivity”. In the middle, there are original contributions addressing more clinical issues such as exploring the possible association of co-existing autoimmunity with the presentation and treatment success in celiac disease and new data on the complex issues of celiac patients who do not respond to a gluten-free diet, either in children with non-responsive celiac disease (NRCD) or adults suspected of refractory celiac disease, where a correct diagnosis ruling out ongoing ingestion of minimal amounts of gluten might be achieved with the use of gluten immunogenic peptides.

The study by Furone et al. (*The protective role of Lactobacillus rhamnosus GG postbiotic on alteration of autophagy and inflammation pathways induced by gliadin in intestinal models*) explores a potential therapeutic option by investigating the possible beneficial effects of a postbiotic (the *Lactobacillus rhamnosus* GG (LGG) postbiotic) in preventing the effects induced by undigested gliadin peptides on the intestinal epithelium. The authors found that this particular postbiotic did indeed prevent the increase of inflammation both in Caco-2 cells and in intestinal organoids from CD patients induced by the gliadin toxic peptide P31-43, thus opening an intriguing door for clinical studies.

The potential effects of autoimmune diseases commonly associated with CD on its overall clinical evolution are examined by the Finnish study by Tauschi et al. (*Association of concomitant autoimmunity with the disease features and long-term treatment and health outcomes in celiac disease*). In a multicenter investigation on more than 800 adult patients, they found that during follow-up on a gluten-free diet, the patients with CD and additional autoimmune conditions experienced significantly poorer general self-perceived health and more overall gastrointestinal symptoms, especially constipation, thus demanding special support.

Approximately 15% of children (1) and up to 30% of adults (2) diagnosed with CD do not show a satisfactory relief of their symptoms within 6–12 months of commencing a GFD. Since, in clinical practice, pancreatic enzyme supplementation is used to alleviate their persisting GI symptoms, Yoosuf et al. (*Pancreatic enzyme supplementation versus placebo for improvement of gastrointestinal symptoms in non-responsive celiac disease: a cross-over randomized controlled trial*) investigated in a prospective, placebo-controlled, double-blind trial the efficacy of pancrelipase co-administered with omeprazole vs. omeprazole only during a 10-day treatment period in adults with NRCD. Their findings, while negative (no significant effect of the pancreatic enzyme supplementation) do offer a clinically useful conclusion.

Non-celiac wheat sensitivity, whose popularity may now be declining (3) has been the object of hot debates in the scientific community, with strong opinions expressed by detractors as well as by supporters of this still unclear and hard-to-diagnose entity. Among the issues at stake is how strict the GFD has to be for these patients (almost exclusively adults) since the documentation of the role of gluten *per se* is unclear (hence, “gluten” is replaced with the better term “wheat”). Seidita et al. in a mostly Italian authored-study (*Potential tolerability of ancient grains in non-celiac wheat sensitivity patients: a preliminary evaluation*) evaluated the frequency of consumption of “ancient grains” and its correlation with clinical manifestations in 233 such patients. In their series, about half of the patients were indeed consuming such grains *before* their NCWS diagnosis, which was associated with a delayed diagnosis, likely due to the better tolerability of these grains, to the point that even *after* diagnosis, a hefty 10% of patients continued their consumption.

A small percentage of adult patients with CD, and especially those with severe and/or prolonged malabsorptive symptoms, may be affected by refractory CD (RCD), a rare condition characterized by a poor prognosis (4, 5). This entity needs to be distinguished from the much more common NRCD (see above), whose most frequent cause remains, both in children and in adults, an ongoing inadvertent gluten ingestion. Thus, assessing dietary adherence is a major component of RCD diagnosis. The Spanish study by Moreno et al. (*Verifying diagnosis of refractory celiac disease with urine gluten immunogenic peptides as biomarker*) utilizes the gliadin immunogenic peptides (GIP) measured in urine (6) to verify dietetic adherence in four patients with RCD, uncovering involuntary gluten ingestion in three of them. Thus, another, if collateral, test is added to support the right diagnosis and proper GFD adherence.

## Author contributions

The author confirms being the sole contributor of this work and has approved it for publication.
